# The Recrystallized Microstructures and Mechanical Properties of a Hypo-Eutectic Al_13_Ni_36_Cr_10_Fe_40_Mo_1_ High-Entropy Alloy

**DOI:** 10.3390/ma18112454

**Published:** 2025-05-23

**Authors:** Hui Li, Han Wang, Xiaoyu Bai, Peng Yan, Linxiang Liu, Chuwen Wang, Yunji Qiu, Zhijun Wang

**Affiliations:** 1 School of Materials Science and Engineering, Chang’an University, Xi’an 710064, China; 2023131021@chd.edu.cn; 2 State Key Laboratory of Solidification Processing, Northwestern Polytechnical University, Xi’an 710072, China; baixiaoyu@mail.nwpu.edu.cn (X.B.); pengy@mail.nwpu.edu.cn (P.Y.); lx_liu@mail.nwpu.edu.cn (L.L.); wangchuwen@mail.nwpu.edu.cn (C.W.); qiuyunji@mail.nwpu.edu.cn (Y.Q.)

**Keywords:** hypo-eutectic high-entropy alloys, recrystallization, microstructure, mechanical properties

## Abstract

Recrystallization is a critical process for tailoring the microstructure to enhance the mechanical properties of alloys. In duplex-phase alloys, the recrystallization is different due to the influence of the second phase. Hypo-eutectic high-entropy alloys (HEAs) with two phases are promising structural materials. Understanding the laws of microstructure and mechanical properties during recrystallization is essential for fabrication and application. Here, we systematically investigate the influence of recrystallization time on the microstructure and mechanical properties of an as-cast hypo-eutectic high-entropy alloy (HEA), Al_13_Ni_36_Cr_10_Fe_40_Mo_1_. As the recrystallization time increases from 10 min to 8 h at 1100 °C, the cold-rolled alloy gradually completed the recrystallization process with a residual large B2 phase and equiaxed FCC grains decorated with B2 precipitation. The average grain size of the FCC phase increases slightly from 2.60 μm to 3.62 μm, while the fine B2 phase precipitates along the FCC phase’s grain boundaries. This optimized microstructure significantly improves the alloy’s tensile strength from 422 MPa to 877 MPa, while maintaining a substantial plasticity of 41%, achieving an excellent strength–ductility balance. These findings provide useful information for regulating the industrial thermomechanical treatment of dual-phase hypo-eutectic high-entropy alloys.

## 1. Introduction

Recently, high-entropy alloys have attracted significant attention owing to their distinctive properties and vast potential applications [1,2,3,4]. The synergistic effects of multiple elements in these alloys lead to exceptional characteristics, such as low density, corrosion resistance [5,6], and high strength combined with plasticity [7,8,9], making them highly promising for a wide range of industrial uses. Among these alloys, the newly developed FCC/B2 dual-phase structure in high-entropy alloys combines the advantages of both the FCC and BCC phases [10,11] and enable broader performance optimization. This microstructure offers an effective approach for attaining an optimal balance between strength and plasticity.

Among the high-entropy alloys, the Co-free AlCrFeNi alloy has emerged as a potential material for industrial applications due to its low production cost and excellent mechanical properties [12,13,14]. Recent studies confirm that adding alloying elements can considerably enhance the mechanical properties of these alloys by modulating their microstructure. For example, microalloying Al_17_Cr_10_Fe_37_Ni_36_ with Mo has been found to improve both yield strength and fracture elongation [15]. Additionally, processing techniques such as cold rolling after annealing [16,17,18], warm rolling [19,20,21], and heat treating [22,23] also markedly impact their comprehensive mechanical properties. Qiu, for instance, used an Al_17_Cr_10_Fe_36_Ni_36_Mo_1_ alloy in its hot-rolled form, followed by annealing and double-stage aging, which resulted in a high tensile strength of 1365.7 ± 9.5 MPa and a fracture elongation of 14.2 ± 1.5% at room temperature [24]. Eshed [13] investigated an AlCrFe_2_Ni_2_ alloy under various heat treatments processes and discovered that acicular B2 phases precipitated within the FCC matrix after solid-solution treatment, and the B2 precipitates further expanded and coarsened after aging.

Although significant advancements have been made in understanding the microstructure and toughening characteristics of AlCrFeNi system alloys [9,25,26,27], the influence of recrystallization processes on these alloys remains insufficiently explored. Among the alloys in the AlCrFeNi system, the novel industrially produced cast Al_13_Ni_36_Cr_10_Fe_40_Mo_1_ hypo-eutectic high-entropy alloy is a promising structural material. Therefore, this paper aimed to study the effect of varying recrystallization times on the Al_13_Ni_36_Cr_10_Fe_40_Mo_1_ alloy. The microstructures and mechanical properties were analyzed in detail to clarify the impact of recrystallization time. This work will contribute to optimizing the heat treatment process for the alloy and provide crucial guidance for its industrial application, particularly for dual-phase hypo-eutectic high-entropy alloys.

## 2. Materials and Methods

### 2.1. Material Preparation

In a vacuum induction melting furnace, chromium, aluminum, nickel, and industrial pure iron (purity ≥ 99.95%) were added in proportion, followed by the addition of a nickel–molybdenum alloy at a certain ratio to manufacture Al_13_Cr_10_Fe_40_Ni_36_Mo_1_ ingots. Afterward, the vacuum induction ingot was additionally treated using electroslag remelting (ESR). After the ESR process, a ϕ 400 mm diameter electroslag ingot was obtained by cooling in air. The electroslag ingot samples were then partially cut into three parts: top, middle, and bottom. The bottom samples were further cut into squares of 60 mm × 60 mm × 20 mm and placed into a muffle furnace (Hefeiikejing, KSL-1400X, Hefei, China,) for homogeneous heat treatment under atmospheric conditions (temperature of 25.5 °C, relative humidity of 60.5%) at 1200 °C for 4 h. Afterward, the samples were rolled in multiple passes at room temperature using a DY-10PYA double-roll mill, maintaining the same rolling direction for each pass and applying a downward pressure of 0.1 mm until the alloy was rolled to 3 mm thickness (70% reduction). To study the recrystallization behavior of the alloy, the samples were annealed in a muffle furnace (Hefeiikejing, KSL-1200X, Hefei, China) at 1100 °C for 10 min, 1 h, 2 h, 4 h, and 8 h. All heat treatment experiments were conducted with air cooling to prevent the occurrence of cracks.

### 2.2. Alloy Testing and Characterization

Cuboid samples with a size of 10 mm × 10 mm × 5 mm were taken at the ingot position of the annealed samples by the electro-spark wire-electrode cutting method, then the samples from different heat treatment states were sanded and polished with 240, 800, 1500, 2500, and 4000-grit SiC papers to remove the surface oxide layers. The polished samples for microstructure characterization were further subjected to electro-polishing in a mixed electrolyte consisting of 90% anhydrous ethanol and 10% perchloric acid at a DC voltage of 30 V for 4–6 s at room temperature. The microstructure and fracture morphology of the solid-solution treatment and the various heat treatment alloys were examined using an optical microscope (OM) (OLYMPUSOLS4000, Tokyo, Japan) and scanning electron microscopy (SEM) (TESCANMIRA3, Brno, Czech Republic) in secondary electron mode. Electron backscatter diffraction (EBSD) (TESCAN MIRA3, Brno, Czech Republic) was applied to further analyze the grain orientation and grain size of the alloys under various heat treatment conditions. The EBSD measurements were carried out using an accelerating voltage of 20 kV with a scanning step size of 0.5 μm, and the data were processed using AZtecCrystal 2.1.2 software. In this study, room temperature (20 °C) tensile tests were carried out using a universal testing machine (UTM, TSMT EM6, Shenzhen, China) at a constant tensile strain rate of 10^−3^ s^−1^. The dog-bone-shaped samples had scaled dimensions of 12.5 mm × 3.0 mm × 2.0 mm, and the strains were accurately measured using a mechanical tensiometer (Sanjing Y12.5/5, Guangzhou, China) with a scale distance of 12.5 mm for the room temperature tensile tests.

## 3. Results

### 3.1. Initial Morphology

**Figure 1:**
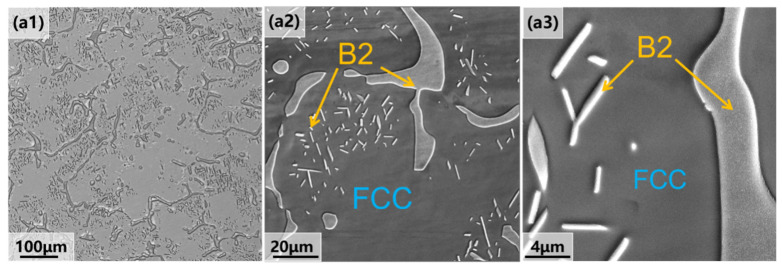
Microstructure characterization of Al_3_Ni_36_Cr_10_Fe_40_Mo_1_ alloy in solid-solution state: (**a1**) optical microscopy image; (**a2**,**a3**) scanning electron microscopy images. To the right of each image is displayed a corresponding magnified view of the alloy’s microstructure.

Figure 1 illustrates the microstructures of the cast Al_13_Ni_36_Cr_10_Fe_40_Mo_1_ alloy after the homogenizing heat treatment. As shown in the light microscope images (Figure 1(a1)), the alloy exhibits a characteristic two-phase structure, with the black regions representing the B2 phase and the gray regions corresponding to the FCC phase. To further investigate the microstructural changes during processing, scanning electron microscopy (SEM) was employed. The as-cast alloy, after homogenizing heat treatment at 1200 °C for 4 h (Figure 1), exhibits a dendritic microstructure with coarse grains and noticeable non-uniformity. This microstructure leads to cracking during processing, thereby limiting its suitability for high-performance applications.

### 3.2. Microstructure and Phases

**Figure 2:**
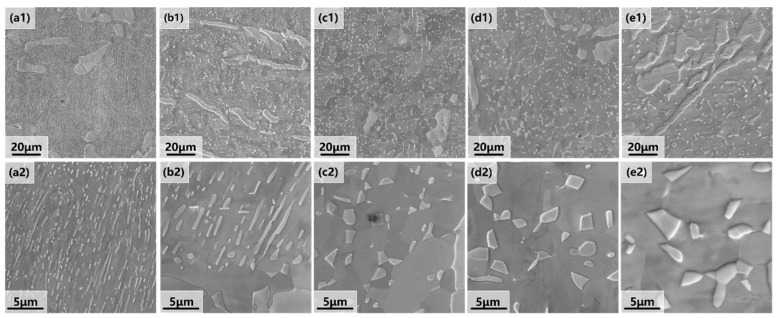
SEM images of the hypo-eutectic high-entropy Al_13_Ni_36_Cr_10_Fe_40_Mo_1_ alloy annealed at 1100 °C for different times: (**a1**,**a2**) annealing for 10 min; (**b1**,**b2**) annealing for 1 h; (**c1**,**c2**) annealing for 2 h; (**d1**,**d2**) annealing for 4 h; (**e1**,**e2**) annealing for 8 h. Below each image is an enlarged image for the corresponding heat treatment time.

Figure 2 presents the SEM images of the alloy recrystallized at 1100 °C for different times, revealing five distinct microstructural states. At the initial annealing stage (10 min), the microstructure consists of both the FCC phase and the B2 phase. Most of the B2 phase exhibits bar-like deformed grains, while a smaller fraction exhibits equiaxed grains (Figure 2(a1)). When the annealing time rises from 10 min to 2 h, the two phases gradually transition from deformed grains to fully equiaxed grains (Figure 2(c1,c2)). Extending the annealing time further to 4 h and 8 h results in grain coarsening in both the FCC and B2 phases. Additionally, fine B2 phases continue to precipitate throughout the process.

### 3.3. Mechanical Properties

**Figure 3:**
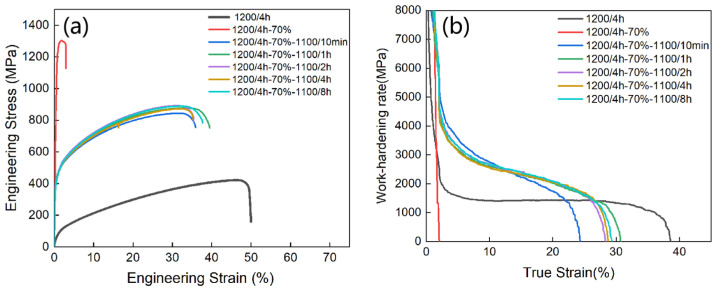
Stress–strain graphs (**a**) and work-hardening rate curves (**b**) of Al_13_Ni_36_Cr_10_Fe_40_Mo_1_ heat-treated at 1100 °C for different times, measured at room temperature.

Figure 3 presents the engineering stress–strain curves and the work-hardening rate graph for the alloy at various annealing times. As shown in the figure, the alloy exhibits excellent plasticity after solid-solution homogenization, with a total plasticity of about 49.9%, but its tensile and yield strengths remain low. After rolling, the yield strength increases significantly, but plasticity drops drastically, with fracture elongation decreasing to 2.9% and the work-hardening capacity nearing zero. These results indicate that neither the solid-solution treatment nor the cold-rolling process alone achieves an optimal strength–plasticity balance.

To address this issue, the alloy was subjected to recrystallization annealing after rolling. As shown in the stress–strain graph, the recrystallization process effectively enhances the strength–plasticity balance of the alloy. When the annealing time was extended from 10 min to 8 h, it was shown that the stress–strain curves of the five HEAs showed little difference. According to the tensile property data summarized in Table 1, the alloy achieves an optimal balance of strength and plasticity after 1 h of annealing, with a yield strength of 426 MPa, a tensile strength of 877 MPa, and a total plasticity of 41%. This improvement is attributed to the different plastic strains of the complex B2 phase and the softer FCC phase during tensile deformation, creating a strain gradient that promotes heterogeneous deformation-induced hardening, enhancing strength while maintaining good plasticity (εp of 41%) [28,29].

## 4. Discussion

### 4.1. Grain Size Variation

**Figure 4:**
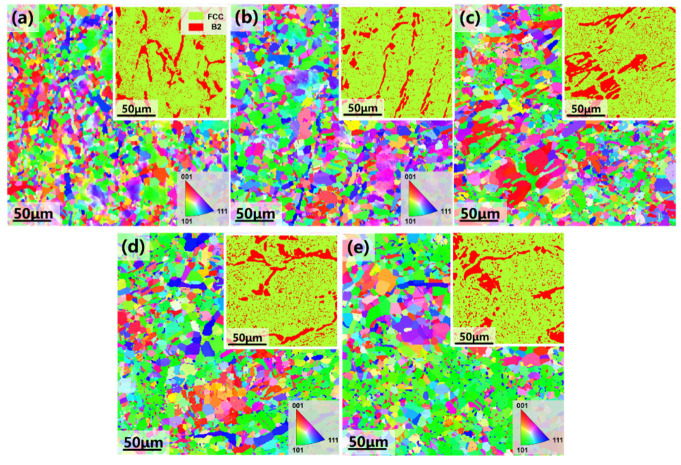
Electron backscattering diffraction (EBSD), grain orientation, and two-phase organization in the recrystallization region of the hypo-eutectic high-entropy Al_13_Ni_36_Cr_10_Fe_40_Mo_1_ alloy heat-treated at 1100 °C for different times: (**a**) 10 min; (**b**) 1 h; (**c**) 2 h; (**d**) 4 h; (**e**) 8 h.

**Figure 5:**
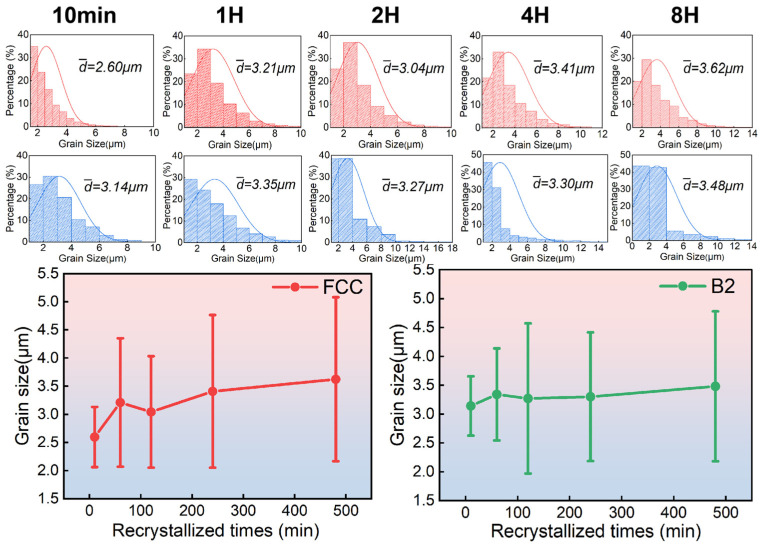
Grain size distribution in the recrystallization region of the hypo-eutectic high-entropy Al_13_Ni_36_Cr_10_Fe_40_Mo_1_ alloy heat-treated at 1100 °C for different times: 10 min, 1 h, 2 h, 4 h, 8 h. The two figures below show the linear plot of the average grain size of FCC phase and B2 phase against recrystallization time. The average grain size image of the FCC phase is in red, and the average grain size image of the B2 phase is in blue.

Figure 4 presents the electron backscatter diffraction (EBSD) results, including grain orientation maps (IPF diagrams), phase distribution maps, and the average grain sizes in the recrystallized regions of the alloy annealed for different annealing durations. The phase distribution maps indicate that the alloy consists of two phases: the B2 phase (red) and the FCC phase (yellow). As the annealing time rises from 10 min to 1 h, the volume fraction of recrystallization gradually increases, while the deformed grains transform into equiaxed shapes. Simultaneously, slight coarsening of both the FCC and B2 phases is observed in the recrystallized regions. The average grain size of the FCC phase increases from approximately 2.60 μm to 3.21 μm, while the grain size of the B2 phase grows from 3.14 μm to around 3.35 μm (Figure 5).

After 2 h of heat treatment, the alloy reaches a fully recrystallized state, exhibiting a lower average grain orientation difference in the recrystallized zone. Both the FCC and B2 phases display completely equiaxed grains, with the equiaxed FCC grains decorated with B2 precipitation (Figure 4c). As the annealing time is extended to 4 h and 8 h, additional fine B2 phase precipitates form, consistent with the SEM observations. The average grain size of the B2 phase increases slightly from approximately 3.27 μm at 2 h to 3.48 μm at 8 h. According to the EBSD data (Figure 5), the average grain sizes of the FCC and B2 phases reach their maximum values after 8 h of annealing, with the FCC phase averaging around 3.62 μm and the B2 phase averaging 3.48 μm.

### 4.2. Strengthening Mechanisms

Since the complex phase constituents of the Al_13_Ni_36_Cr_10_Fe_40_Mo_1_ HEA consist of the FCC phase and B2 phase, the mixing rule was applied to evaluate its yield strength (YS, $\sigma_{0.2}$) [30]

(1) $$\sigma_{0.2}=\sigma_{0}+{∆\sigma }_{s}+{∆\sigma }_{G}+{∆\sigma }_{P}$$

where the $\sigma_{0}$ is the lattice friction strength (herein, 254 MPa was used for the Al_13_Ni_36_Cr_10_Fe_40_Mo_1_ HEA by referring to the Fe_50_Ni_25_Cr_15_Al_10_ HEA [31]), and ${∆\sigma }_{s}$, ${∆\sigma }_{G}$, and ${∆\sigma }_{P}$ are the strengthening contributions of the solid solution, grain boundaries, and precipitates, respectively.

The solid solution strengthening of Al_13_Ni_36_Cr_10_Fe_40_Mo_1_ HEA can be simplified as a non-equiatomic NiCrFeAl solvent with a Mo solute. In this study, we used the 60 MPa to roughly evaluate the solid solution strengthening of the FCC phase [15].

The grain boundary strengthening effect can be quantitatively described by the classical Hall–Petch relationship [32]

(2) $${∆\sigma }_{G}=kd^{-\frac{1}{2}}$$

where k is the Hall–Petch coefficient (herein, 226 MPa·μm^1/2^ [31] was applied for the FCC phase, and 302 MPa·μm^1/2^ [33] was the B2 phase), and *d* is the grain size. For the Al_13_Ni_36_Cr_10_Fe_40_Mo_1_ HEA, the average sizes and volume fractions of the precipitates are listed in Table 2.

The precipitation strengthening mechanism originates from two distinct contributions: dislocation shearing of precipitates and Orowan bypassing. For precipitates exceeding a critical size, the Orowan mechanism becomes dominant, which can be quantitatively described as [34]

(3) $${\Delta \sigma }_{Orowan}=M\;\frac{0.4Gb}{\pi \sqrt{1-\vartheta }}\frac{\ln\left(\frac{2r}{b}\right)}{2r\left(\;\sqrt{\frac{\;\pi \;}{4f}}-1\;\right)}$$

where G is the shear modulus, M is a Taylor factor (2.73 for BCC, 3.06 for FCC), ϑ is the Poisson ratio (ϑ = 0.314), b is the Burgers vector (b = $\frac{\sqrt{3}a}{2}$ for BCC, b = $\frac{a}{\sqrt{2}}$ for FCC, a is the lattice parameter), f is the volume fraction, and r represents the mean radius of precipitates measured from their circular cross-sections on random planes.

According to Equations (1)–(3), $\sigma_{0}$, ${∆\sigma }_{s}$, ${∆\sigma }_{G}$, and ${∆\sigma }_{P}$ are 254, 44.4, 136.24, and 12.38 MPa, respectively. So, the sum $\sigma_{0.2}$ was calculated to be 447.03 MPa, which shows reasonable consistency with the experimental measurement (426 MPa, 1100 °C/1 h annealing). The observed reason primarily stems from statistical uncertainties associated with the limited sampling area, particularly affecting the determination of the phase volume fraction and the precipitates’ dimensions.

### 4.3. Fracture Surface

**Figure 6:**
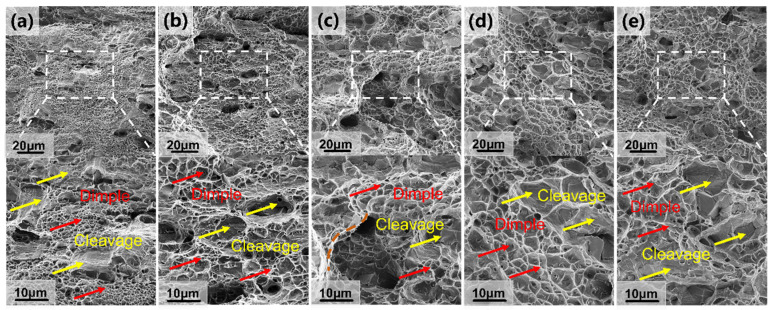
Secondary electron micrographs of the fracture morphology of the Al_13_Ni_36_Cr_10_Fe_40_Mo_1_ alloy annealed at room temperature for different times at 1100 °C: (**a**) 10 min; (**b**) 1 h; (**c**) 2 h; (**d**) 4 h; (**e**) 8 h. The inset shows the macroscopic fracture of the corresponding alloy. The brown lines in the figure represent large-scale tearing edges formed at the interfaces between dissociated surfaces and ligamentous nodular structures.

To further analyze the fracture behavior of the alloy at different annealing times, secondary electron characterization was employed to examine the tensile fracture morphology at room temperature. As shown in Figure 6, all alloys display two typical structural features: disintegrated surfaces and ligamentous nests. The brittle B2 phase primarily fractures through disintegration, while the softer FCC phase, with more slip systems, fractures mainly through ligamentous nests. After recrystallization annealing, the fracture surface exhibits more small-sized ligamentous nests, indicative of a typical ductile fracture mode. Although the fracture mechanisms remain consistent across different annealing times, the size of the ligamentous nests increases significantly when the annealing time reaches 1 h. Additionally, the presence of pits within the fracture morphology suggests that the B2 phase is distributed within the FCC grains, which aids in restoring plasticity and inhibiting crack initiation and propagation. When the annealing time is extended to 2 h, large tearing edges appear at the interface between the disintegrated surfaces and ligamentous nests (Figure 6c). Further extending the annealing time to 4 h and 8 h leads to further growth of the ligamentous nests, with a significant increase in the fraction of disintegrated surfaces, indicating ongoing precipitation of the B2 phase at the FCC grain boundaries and coarsening.

## 5. Conclusions

This paper applies mechanical techniques including cold rolling and high-temperature annealing to the cast Al_13_Ni_36_Cr_10_Fe_40_Mo_1_ alloy, with systematic investigation of the microstructural and mechanical properties’ evolution mechanisms in the alloy during recrystallization. The as-cast alloy exhibited a dendritic structure, consisting of both the FCC and B2 phases. As the annealing time was prolonged from 10 min to 8 h, recrystallization progressed gradually. Compared with the initial microstructure, the FCC and B2 phases transformed from elongated deformed grains to equiaxed crystals after recrystallization, accompanied by the precipitation of fine B2 phase particles along the FCC grain boundaries. The average grain sizes of the FCC and B2 phases increased slightly from 2.60 μm and 3.14 μm to 3.62 μm and 3.48 μm, respectively, after 8 h of annealing.

Furthermore, the alloy’s strength and plasticity did not show significant changes during recrystallization. However, the optimized microstructure led to a marked enhancement in the alloy’s yield strength, reaching 426 MPa, and tensile strength, reaching 877 MPa, after 1 h of annealing, while maintaining a substantial plasticity of 41%. This achieves an excellent strength–ductility balance. These improvements are attributed to the cooperative interactions of solid solution strengthening, grain boundary strengthening, and B2 phase precipitation strengthening. This work provides useful information for tailoring the microstructure and properties of dual-phase hypo-eutectic high-entropy alloys through controlled recrystallization. The identified annealing parameters (1100 °C, 1 h) offer a reproducible approach to achieving both high strength and ductility, thereby advancing the industrial application of these alloys.

## Figures and Tables

**Table 1:** Tensile properties of Al_13_Ni_36_Cr_10_Fe_40_Mo_1_ heat-treated at 1100 °C for different times.

	σ_0.2_, MPa	σb, MPa	εp, %
1200 °C/4 h	70.5	422.2	49.9%
1200 °C/4 h-70%	1178.7	1302.6	2.9%
1200 °C/4 h-70%-1100/10 min	423	844	34.5%
1200 °C/4 h-70%-1100/1 h	426	877	41%
1200 °C/4 h-70%-1100/2 h	415	892	35.4%
1200 °C/4 h-70%-1100/4 h	365	873	36.5%
1200 °C/4 h-70%-1100/8 h	392	889	39.5%

**Table 2:** The average size and volume fraction of precipitates.

Precipitates	Average Size (μm)	Volume Fraction (%)
FCC	3.21	74
B2	3.35	26

## Data Availability

The original contributions presented in this study are included in the article. Further inquiries can be directed to the corresponding authors.
